# Introduction to special issue, “How nature shaped echolocation in animals”

**DOI:** 10.3389/fphys.2013.00193

**Published:** 2013-07-24

**Authors:** Cynthia F. Moss, Mariana L. Melcón

**Affiliations:** ^1^Psychology Department and Institute for Systems Research, University of MarylandMD, USA; ^2^Scripps Institution of Oceanography, University of CaliforniaSan Diego, CA, USA

This special issue, “How nature shaped echolocation in animals,” is dedicated to Elisabeth Kalko and Björn Siemers, two extraordinarily creative, passionate, and important researchers in the field of echolocation. Both Eli and Björn passed away suddenly and at young ages, leaving a gaping hole in our research community. Eli, with enormous talents as a naturalist and a contagious enthusiasm to understand the lives of animals in the field, broke new ground in her discoveries of the diversity and richness of bat behaviors. Björn combined exceptional imagination and scientific rigor to make keen observations on bat echolocation and to launch a world class program combining laboratory and field studies.

**Figure 1:**
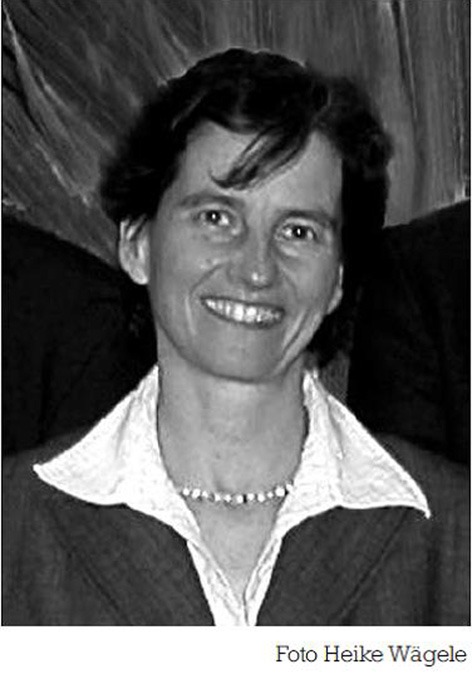
**Elisabeth Kalko. April 10, 1962 to September 26, 2011**.

**Figure 2:**
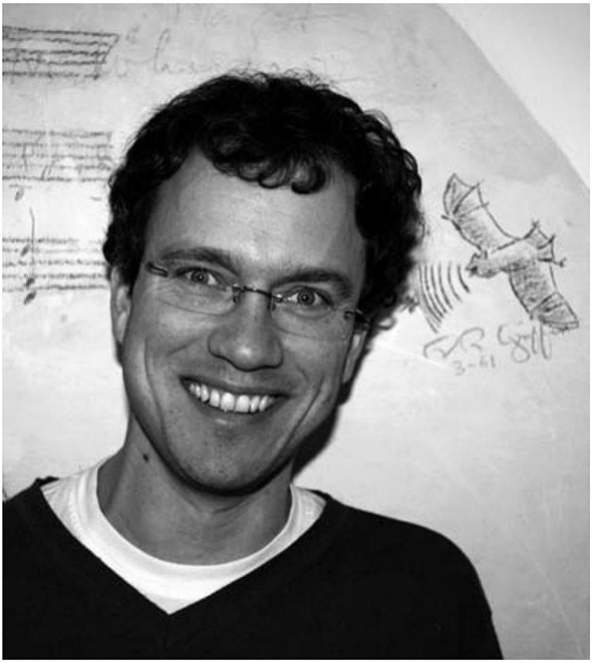
**Björn Siemers (May 25, 1972 to May 23, 2012) in front of the “autograph wall” in the former Von-Holst-House in Seewiesen**. In the background is a bat drawn by Donald Griffin in 1961.

For those who did not have the chance to meet Elisabeth Kalko (see Figure 1) and Björn Siemers (see Figure 2), we hope that the obituaries below will give you some understanding of their contribution and personalities. And for those who had the pleasure of knowing them, this is just another way to remember them.

Professor Dr. Elisabeth Kalko, Director of the Institute of Experimental Ecology at the University of Ulm, died most unexpectedly on September 26, 2011, at the age of 49. She died in her sleep during a research trip to Mount Kilimandjaro in Tanzania. As the website of the Smithsonian's Tropical Research Center in Panama City put it, she passed away while doing what she loved most: research with bats.

Elisabeth Kalko was born in Berlin on April 10, 1962. After receiving her “Abitur” [German higher education entrance certificate; preparatory school diploma] from the Justinus-Kerner Gymansium in Heilbronn she began her studies in biology at the University of Tübingen in the fall of 1981 and completed a Master's degree in 1987. From 1984 until completing her PhD, she held a scholarship from the German National Academic Foundation.

With her MS project in animal physiology in Tübingen, “Hunting and Echolocation Behavior of the Daubenton's bat, *Myotis daubentonii*, in the wild,” Kalko had already discovered the research area that would be the focus of her future career.

In 1991, Kalko completed her doctoral work on “The echolocation and hunting behavior of three European species of common pipistrelles, *Pipistrellus pipistrellus, P. nathusii, P. kuhlii*, in the wild” and graduated summa cum laude in Tübingen. Her work was honored with the Fritz Lang Prize of the German Society for Mammalian Biology (DGS).

The publications resulting from Kalko's graduate research set new standards for conducting field work on bat echolocation and are frequently cited to this day. Because of the high quality of this work, Kalko quickly became internationally renowned. Everyone spoke highly of her unique ability to capture bat behavior with tireless energy, great patience, an intuitive understanding of animal behavior, and an extraordinary empathy for nature. This quickly made her an important figure in bat research. Kalko's extraordinary impact is reflected in a statement by Donald Griffin, the discoverer of bat echolocation: After she enthusiastically showed him her field data, he spontaneously called her the “Jane Goodall of bats.” Coming from Griffin, a critical scientist who was known to express praise sparingly, this was especially noteworthy recognition.

After she received her doctoral degree and during the postdoctoral phase of her career, Kalko was able to pursue her research tirelessly and with great success through two DFG-[German Research Foundation]-funded projects, “Diversity in Tropical Bats: Resource Utilization, Habitat Selection, and Niche Specializations of a Tropical Species Community” and “Comparative Studies on the Organization, Structure, and Dynamics of Neotropical Bat Communities in Disturbed and Undisturbed Forest Systems.” These research topics also formed the basis for her Heisenberg-funded habilitation work, which she completed at the University of Tübingen in 1999 with a thesis on “Diversity, Structure and Dynamics of Neotropical Bat Communities.”

Even before she completed her habilitation, Kalko was awarded a professorship in Experimental Ecology at the University of Ulm. At the same time she was promoted to staff scientist at the STRI in Panama. The good working conditions in Ulm and Panama enabled Kalko to build a highly productive research group and to considerably broaden her field of interest and research, as described in an excerpt on her University of Ulm website:

“My research focuses on community ecology, sensory ecology, behavioral ecology, ecophysiology and diversity patterns of vertebrate assemblages, particularly in the species-rich tropics with a focus on bats (Chiroptera). I am particularly interested in functional diversity and the effects of changes in land use and climate change on biodiversity patterns and ecosystem services with the ultimate goal to feed the results of my studies into applied sciences, particularly into conservation biology and into the emerging field of zoonotic diseases with regard to wildlife and human health. Another focus of my research deals with sensory systems of bats with special emphasis on ecological and evolutionary aspects of their echolocation system and foraging strategies integrating multiple sensory cues, i.e., olfaction and vision. As a third cornerstone of my research I am concentrating on bat-plant interactions, particularly frugivory and the adaptations between consumers/dispersers and plant traits. My study areas cover temperate zones, particularly Europe, and the tropics, mainly Central- and South America (Panama, Costa Rica, Mexico, Venezuela, Bolivia, Peru) and Africa (Tanzania, Benin, Ivory Coast, Ghana).”

The great success of Kalko's work is evident in the many academic honors she received, such as her election to the National Committee of Global Change in Germany in 2002 and to the Heidelberg Academy of Sciences [HAW] in 2004. In 2005, she became vice president of the Society for Tropical Ecology [GTÖ], followed in 2008 by her election as a member of the Senate Commission on Biodiversity Research of the German Research Foundation [DFG], and as chair of DIVERSITAS Germany. In 2011, shortly before her death, she became a member of the University Council at the University of Ulm.

Kalko was a talented teacher and received the State Teaching Award of Baden-Wüttemberg. She captured the attention of her audience with her great expertise in behavioral ecology and her enthusiasm for nature, making a lasting impression on the listener. This ability was well-known at the STRI in Panama. Whenever important politicians or VIPs arrived from Washington, Kalko was asked to guide them through the forest and discuss her research, which inevitably had favorable effects on future research funding. Everyone who experienced Kalko in the field will certainly agree that no one could convey biological knowledge, love for nature, and amazement about the natural world better than she did.

Elisabeth Kalko had great fervor for her work, was passionately devoted to science, and viewed her research as both a job and a calling. She launched many projects, positively influenced scientists and students, and touched them with her engaging personality. German Zoology has experienced a great loss with her passing. We deeply miss her.

—Prof. Dr. H.-U. Schnitzler and Dr. A. Denzinger

On May 23, 2012, Assistant Professor Dr. Björn Martin Siemers died as a result of an infection, within only a few hours, and just two days before his 40th birthday. His death came as a great shock to his wife and two children, his family, his workgroup at the Max Planck Institute for Ornithology in Seewiesen, and to his many students, colleagues, and friends.

Björn Siemers was born in Stuttgart on May 25, 1972. Early on in his life it was already clear that he would become a natural scientist, as there was nothing he enjoyed more than imaginary research expeditions with his brother. After his “Abitur” [German higher education entrance certificate; preparatory school diploma] he studied animal physiology, zoology, and genetics, combined with a minor in law at the University of Tübingen. In 1994–1995 he was a visiting student at the University of Sao Paulo in Brazil for study abroad, funded by the DAAD [German Academic Exchange Service], where he studied primatology, entomology, and neurobiology. Despite his enthusiasm for primates and his decision to become a primatologist, when he returned to the University of Tübingen he began working with bats as part of his major practical lab course (“Grosspraktikum”) in animal physiology. Bat research continued to impact his career development and resulted in important discoveries during his master's and doctoral work and in many subsequent investigations. However, his research profile and his scientific collaborations over the past few years show that he never fully abandoned primatology.

Siemers's graduate work laid the foundations for his primary area of research: sensory ecology. In his master's project he investigated the hunting and echolocating behavior of the Natterer's bat and showed for the first time that these bats are able to locate their prey even if the prey is positioned very close to background clutter. For his doctoral work he expanded his research by conducting comparative studies of prey perception in different species of *Myotis*, completing his research in 2000. He demonstrated that the echolocation calls of the different *Myotis* species, which actively find their prey using echolocation, are characterized by species-specific differences, particularly in their call bandwidth. This can be interpreted as an adaptation to habitat-specific echolocation tasks. The wider the species-specific signal bandwidth, the smaller the separation a bat could detect between prey and background. These experimental studies were the first to confirm a previously untested hypothesis derived from sonar theory. Subsequent publication of the results in *Nature* earned Siemers international acclaim. In the 5½ years following his doctoral degree he worked as Assistant Professor in animal physiology and further qualified himself with additional studies on the sensory ecology of prey perception in bats and primates, work which cumulatively led to his habilitation at the University of Tübingen in 2006. Due to his extraordinary scientific achievements and approaches, immediately after his habilitation he was selected for a position as “Independent Young Scientist” at the Max Planck Institute, after a highly competitive selection process. This is a particularly great achievement, since only 2% of the applicants were selected. With his usual dynamism and great enthusiasm he began building a “sensory ecology” research group at the Max Planck Institute for Ornithology in Seewiesen in the summer of 2006. This research group focused on comparative studies of sensory and cognitive specializations for foraging in animals, and the resulting niche differentiation.

At the time of his death, Siemers's group consisted of 15 members, with whom he conducted research both nationally and internationally. Siemers's great productivity resulted in many high quality publications in top tier journals, such as *PNAS* and *Nature*. Also impressive was his ability to convey his broad knowledge of bats beyond the halls of academia. The books he published in collaboration with the well-known animal photographer Dietmar Nill, *Fledermäuse—Das Praxisbuch* [*Bats—A Practical Guide*] and *Fledermäuse. Eine Bilderreise in die Nacht* [*Bats. A Photographic Journey into the Night*], are an excellent introduction to the lives of these animals. All aspects of a bat's life are made comprehensible, with exciting narratives and surprising facts, and important species are introduced in brief portraits. With a strong media presence, Siemers impressed his audience with extraordinary stories and excellent photographic and video material.

In 2009, Siemers and colleagues from Israel and the LMU in Munich received $900,000 in funding from the Human Frontiers Science Program for a project entitled “Listening through the Looking Glass: Perception and Neural Encoding of Mirror Images of Biosonar.” In 2011, Siemers was awarded the prestigious “European Starting Grant” in the amount of 1.5 Million Euros from the European Research Council on the topic, “Sensory and Cognitive Ecology of Interspecific Interactions in Bat Communities.” Considering these many successes, a full professorship seemed only a question of time.

With the untimely death of Siemers, German Zoology loses not only a promising scientist but also an exceptional and kind human being. Siemers's curiosity, eagerness to engage in discussions, willingness to help others, and cheerfulness were contagious and inspired everyone. Anyone greeted by Siemers in the morning with his cheery “Good Morning” would be hard pressed to start the day in a bad mood. His optimism and positive attitude were astounding. Though his disability might have given him reason to bear a grudge against fate, he radiated vital energy and optimism and took delight in his work and life. He accepted life in its entirety. He believed in the good in every person and also in institutions, and made a positive impact with this attitude. These qualities made Siemers a popular teacher as well. He always listened to his students with a sympathetic ear. They cherished him for it and felt accepted. His humor, too, was quite endearing, and like almost nobody else he was able to laugh at himself—even when we were amused by his often [overly] professorial statements.

Björn Siemers focused his life's priorities not only on his career and work, but also—and always—on his family and children. Siemers will live on in our thoughts and be with us forever.

—Dr. A. Denzinger and Prof. Dr. H.-U. Schnitzler

Both obituaries translated by Dr. S. Blumenrath and reprinted with permission of the authors. Original published in ZOOLOGIE 2012, Mitteilungen d.Dtsch.Zool.Ges.

